# Spatial Learning of Individual Cichlid Fish and Its Effect on Group Decision Making

**DOI:** 10.3390/ani12101318

**Published:** 2022-05-21

**Authors:** Jiaxing Long, Shijian Fu

**Affiliations:** Chongqing Key Laboratory of Animal Biology, Chongqing Normal University, Chongqing 401331, China; 2020110513015@stu.cqnu.edu.cn

**Keywords:** cichlids, memory retention, learning, six-arm maze, group decision

## Abstract

**Simple Summary:**

Learning and memory abilities and their roles in group decision-making have important ecological relevance in routine activities such as foraging and anti-predator behaviors in fish species. The aims of this study were to explore the spatial learning ability of juvenile cichlids (*Chindongo demasoni*), and the influence of heterogeneity of memory information among group members on group performance in a six-arm radiation maze. The spatial performance of individual fish improved and reached a stable level on the fifth day of training, and the memory of the space task is kept after 11 days of detraining. The spatial performance of heterogeneous groups composed of members with different memory information were found to change linearly with the increase of the proportion of trained members. This indicates that cichlids can obtain associative learning information through training, and it seems unlikely that cichlids’ group behavior is determined by minority members in foraging context.

**Abstract:**

Learning and memory abilities and their roles in group decision-making have important ecological relevance in routine activities such as foraging and anti-predator behaviors in fish species. The aims of the present study were to explore individual spatial learning abilities of juvenile cichlids (*Chindongo demasoni*) in a foraging context, and to explore the influence of heterogeneity of memory information among group members on group performance in a six-arm radiation maze. In the context of an association between landmarks and food, learning ability was evaluated by the speed and accuracy of reaching the arm with food during seven days of reinforcement, and memory retention was tested at intervals of 2, 5, 8 and 11 days of detraining. Then, the speed and accuracy of an eight-member group with different proportions of memory-trained fish were measured. Both speed and accuracy of individual fish improved significantly and linearly in the first five days of training and leveled off between five and seven days, with values 60% shorter (in speed) and 50% higher (in accuracy) compared to those of the first day. Neither speed nor accuracy showed any decrease after 11 days of detraining, suggesting memory retention of the spatial task. When measured in a group, the speed and accuracy of the majority of the group (more than half) in reaching the arm with food changed linearly with an increasing ratio of trained members. This shows that cichlids can acquire associative learning information through a training process, and group behavior of cichlids seems not likely be determined by a minority of group members under a foraging context.

## 1. Introduction

In recent years, research in cognitive science has been largely aimed at the in-depth study of the brain [1,2], but the study of animal learning and memory has also become a focus of attention as an important part of cognitive science [3,4]. Learning and memory are among the most basic functions of the animal brain. The former is the process by which animals acquire new information and knowledge, whereas the latter is the process of saving and reading the acquired information [5]. Associative learning is the learning of an association between two stimuli or events [6,7], whereas operant conditioning is a type of associative learning usually occurring through repeated reinforcement of a certain behavior [8]. Operant conditioning is often investigated in animal individuals placed in diverse devices such as shuttle boxes, T-mazes, or plus mazes using either simple response discrimination (right vs. left) or a visual cue (e.g., reward) marking the correct choice [9]. For example, researchers found that trained fifteen-spined sticklebacks (*Spinachia spinachia*) and corkwing wrasse (*Crenilabrus melops*) can find food more efficiently in radiation mazes by learning through the link between food with visual spatial cues [10].

Foraging behavior is one of the basic behaviors that animals rely on for survival, and the spatial learning and memory of animals (especially those living in complex habitats) can help improve their foraging efficiency, and ultimately, improve the individual’s survival fitness [11,12]. Animals can solve a spatial task by learning an association between a response and a reward (response strategy) or they can learn an association between a given place and a reward (place strategy) [13]. For example, studies in zebrafish (*Danio rerio*) in plus mazes found that trained zebrafish can locate the food arm through association learning [14]. Recently, using T-mazes as arenas, a comparison study in four fish species found a profound difference in spatial learning and memory abilities at both inter- and intra-species levels [15]. More recent studies in cichlids such as *p. taeniatus* found that the spatial learning and memory ability tested in the maze might be altered by rearing condition and gender [16,17,18]. In this experiment, cichlids that usually live in complex habitats were selected as the experimental model, and associative learning between food and a landmark was studied in the arena of a six-arm maze. The first aim of the present study was to test whether operant conditioning could be formed under such conditions by evaluating the speed and accuracy of reaching the landmark arm with food located behind the landmark during a week of training. The second aim of the present study was to test the memory retention of the possible operant conditioning by evaluating the speed and accuracy after different periods (2 to 11 days) of detraining. To the best of our knowledge, this is the first study of operant conditioning through a landmark and foraging behavior to test the memory retention in cichlids in a six-arm radiation maze.

The phenomenon where animals gather in groups and cooperate is called group behavior, which is the survival strategy gradually evolved by animals in the process of natural selection [19,20,21,22]. It has been suggested that communication within the group members speeds up the transmission of information, hence improving cognition ability and increasing the efficacy of foraging [23,24]. An animal group usually needs to make collective decisions when faced with different options while foraging, often deciding between multiple spatially distributed options [25]. Collective decision-making research has received widespread attention in recent years [26,27,28]. Based on these studies, researchers have made certain theoretical breakthroughs in understanding group decision-making mechanisms through mathematical modeling [13]. For example, researchers have proposed two group decision-making mechanisms: meritocratic leadership decisions and consensus decisions. The main feature of the former is that members of the group can disproportionately affect the group decision-making through leadership [7,29]. However, on the contrary, the exchange of information and members’ contributions to the group behavior in a consensus decision appear to be more even and equal [20]. Although important theoretical breakthroughs have been made in group decision-making, the results of investigations in fish species are quite controversial [30,31], and the underlying mechanism needs further investigation. Recently, some researchers proposed a radiative six-arm maze device system, which provides a possible paradigm of discrete alternatives in a series, to be faced by the individuals in an animal group and is an ideal device for group decision-making theory verification and related experiments [7]. Thus, the third aim of this study was to investigate the decision-making mechanism of cichlids by evaluating the speed and accuracy of a fish group comprising different ratios of trained and untrained (i.e., informed and non-informed) members. Because it is difficult to identify individuals within the group, the speed and accuracy of the fish group were evaluated based on the majority (i.e., more than half the members of the group). We anticipated that both variables might improve in groups with a presence of trained fish as a consequence of communication among group members. Furthermore, we assumed that if the speed and accuracy improved profoundly in a group with the presence of only a few trained members but showed less improvement with the increase in trained members, the collective decision making might be affected by a minority of group members. On the contrary, if group performance was linearly correlated with the ratio of informed individuals, the group behavior would be more determined equally within the group members.

*Cichlidae* belong to the class Osteichthyes, of order Perciform. The family is an ideal candidate for fish behavior research [23] as these fish are beautiful in appearance, easy to raise, and have complex social group behaviors [24]. In this experiment, juvenile Demasoni (*Chindongo demasoni*) from Lake Malawi were selected as the experimental animals. The pilot experiment showed that this species possesses a good learning and memory capacity.

## 2. Materials and Methods

### 2.1. Acclimation and Handling of Experimental Fish

The cichlids used in the experiment were purchased in October 2021 from the Mashi Aquarium in Shapingba District, Chongqing. The purchased experimental fish were sterilized with a 1–2% concentration of sodium chloride solution and placed in a self-purification circulating and temperature-controlled rearing system (the volume of each rearing unit was about 20 L; the length was 35 cm, the width was 20 cm and the height was 28 cm) for 14 days, and the water temperature was maintained at 25 ± 0.5 °C during acclimation. The water for acclimation was tap water that was aerated for 24 h. During the acclimation period, air was continuously added to the water with an air pump to ensure that the dissolved oxygen in the water body was maintained above 7 mg L^−1^. Experimental fish were fed with commercial feed once daily at 9:00, and after one hour, the remaining food and fish excrement and feces were siphoned out; the daily water exchange was about 10% of the total water body. After acclimation, 360 healthy juveniles with a mass of 2.52 ± 0.59 g and a body length of 4.60 ± 0.70 cm were selected as the experimental subjects.

### 2.2. Experimental Setup and Behavioral Measurement Protocol

**Figure 1:**
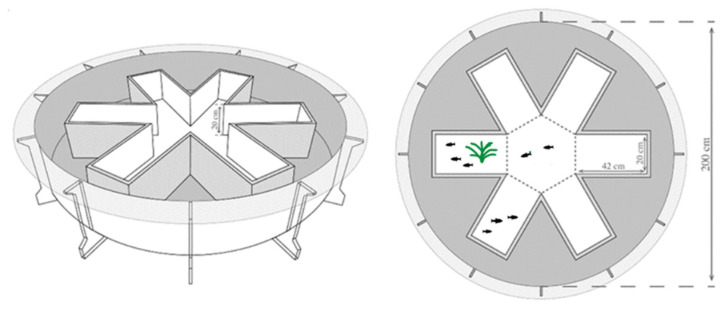
Schematic drawing of the radial six-arm maze used in the present study.

The experimental device was a modified six-arm maze (Figure 1), designed according to earlier studies [25]. Each arm of the device was a 20 cm × 40 cm cuboid structure. The side-length of the regular hexagon in the center was also 20 cm, and the water depth of the maze was 5 cm. The experimental light sources were six fluorescent lamps that ensured the uniform illumination of the shooting site. The plastic plant was used as landmark, i.e., the associative information for food in the present study [32]. During the experiment, an arm was randomly selected, and a plastic plant placed within it. Approximately 5 mg of commercial feed was added to the end of the same arm. In this way, the experimental fish could not directly see the food, and could only reach and consume it after they swam through the plastic plant. The location of food along with the plastic plant was changed each day to avoid the effect of spatial orientation ability on analysis of the results [33,34,35]. Before the start of each experiment, the experimental fish were transferred from the rearing system using an opaque cylindrical adaptor and placed in a blank arm spaced one arm away from the arm with food for 10 min adaptation according to a pilot experiment. We placed a cylinder at the mouth of the arm they were in to prevent the experimental fish from seeing the arm with food in advance. Then, the adaptor was removed, and a camera (LogiCapture 920) placed 1 m directly above the equipment was used for continuous shooting at 15 frames per second for 10 min.

### 2.3. Experimental Scheme

#### 2.3.1. Experiment 1: Training and Memory Test

##### Training Process

Sixty individuals were randomly selected. Each day at approximately 9:00, 12:00 and 15:00, fish were transferred to the maze individually three times. The activity of experimental fish was recorded for 10 min after a 10-min pre-acclimation period (see detail in aforementioned measurement protocol). The training process lasted for seven consecutive days. The arm in which the artificial plants and food were placed was changed daily to avoid the influence of spatial memory on the behavior of the experimental fish [33]. Among the 60 trained fish, 5 individuals showed no improvement in performance (see parameters for detail) and so their training process was terminated between the second and fourth days.

##### Memory Retention Test

After the seven days of training, 48 individuals were randomly selected and the activity was recorded exactly the same as the training process, once again, after 2, 5, 8 and 11 days of detraining (12 repetitions at each time point). After the first training process, the fish were reared individually, meaning each fish could be identified during the training process and memory retention test.

#### 2.3.2. Experiment 2: Group Decision Test

After the memory retention test, heterogeneous trained groups were formed by different ratios of trained and non-trained fish (0:8, 1:7, 3:5, 5:3, 7:1 and 8:0) That is, 0:8 refers to 0 nontrained fish and 8 trained fish, 1: 7 refers to 1 nontrained fish and 7 trained fish, and so on. We conducted eight repetitions for each ratio. Trained fish were used repeatedly after they underwent two rounds of measurement (0:8 to 8:0), whereas nontrained fish were used only once. In brief, eight individuals as a group comprised a specific number of trained or nontrained fish, which were randomly selected. Then, all group members were immediately moved into the second left or right arm next to the arm with food to make sure that the fish could not see the landmark before they swam out of the arm.

The water temperature and other conditions during the experimental tests were the same as those in the acclimation period, and all experimental fish were tested after fasting for two days.

### 2.4. Experimental Parameters

The parameters of the speed and accuracy in reaching the arm with food were used to measure the fish’s cognitive performance. The speed to reach the arm with food was calculated as the period from the beginning of the observation to when the fish went through the plants (observed by the experimenter via video). Accuracy was calculated as the proportion of experimental fish to reach the arm with food the first time (i.e., crossing the landmark) among all fish test members.

Because experimental fish cannot be identified individually, and they seldom form a complete group (i.e., all group members in only one arm), for the group decision-making measurement, a majority group was defined as more than half of the group members (i.e., five fish in the present study) located in any arm of the maze. The speed and accuracy of the majority of the group were calculated exactly the same as we did for individual measurements.

### 2.5. Data, Statistics and Analysis

The data were first inputted to Excel 2003, and then the SPSS 26.0 software was used for statistical analysis. The training effect was tested using a linear mixed model using the fish ID as the random factor and the training days as the main factor. Memory retention was tested by a linear mixed model using the fish ID as the random factor and the detraining period and treatment (seven days trained vs. detrained) as the main factors. The effect of group composition on the speed of the majority in the arm with food was tested by one-way analysis of variance (ANOVA). Then, Duncan’s multiple comparisons were used to analyze the statistical differences between the groups. The relationship between group composition and accuracy for the majority of the group was analyzed by linear regression. The data are expressed as the “mean ± standard error”, and the significance level is *p* < 0.05.

## 3. Results

### 3.1. The Training Effect and Memory Retention of Experimental Fish

#### 3.1.1. Training Effect

**Figure 2:**
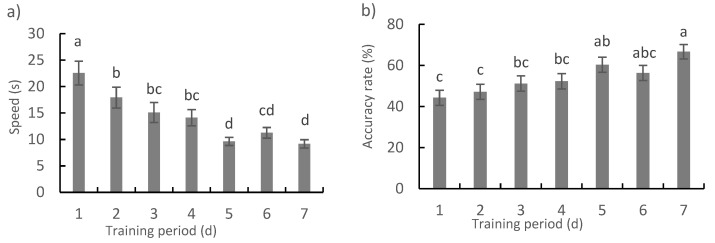
The speed (**a**) i.e., time when fish first arrived at arm with food, and accuracy rate (**b**) i.e., proportion of fish that first entered the arm with food rather than an arm without food, for the foraging task in the maze during the training period (means ± S.E., *n* = 55). Note: a, b, c, d letters suggest a significant difference of the variables after different days of training (*p* < 0.05).

Training had a significant effect on both the speed (*F*_1,57_ = 208.01, *p* < 0.001) and accuracy rates (*F*_1,57_ = 816.53, *p* < 0.001) based on the results of the linear mixed model. The speed, i.e., time taken to first arrive at the arm with food, decreased gradually and significantly from 22.53 s to 9.17 s during the first five days of training (Figure 2a) (*p* < 0.05). However, there was no further decrease from the fifth to seventh days of training. Similarly, the accuracy rate, i.e., proportion of experimental fish to first arrive in the arm with food rather than arm without food, increased from 44.25% (which is much higher than 13.3%, the theoretically calculated value of one-sixth) to 60.34% during the first five days of training (there was no significant difference in the first four days, although the values gradually increased) (Figure 2b) (*p* < 0.05), whereas there was no further increase from the fifth to seventh days of training.

#### 3.1.2. Memory Retention

Neither speed nor accuracy rate of fish after the seventh day of training and those with 2 to 11 days of detraining showed any significant difference (Table 1).

### 3.2. The Effect of Group Composition on the Decision Making of Experimental Fish

**Figure 3:**
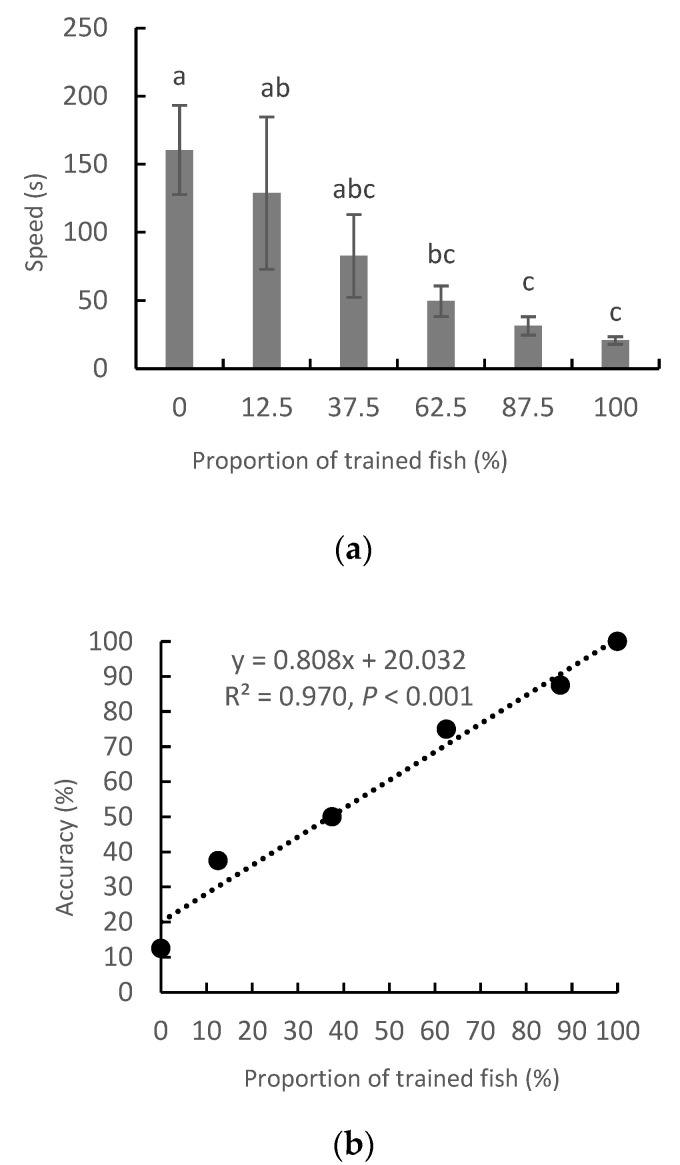
The speed (*n* = 8) (**a**) and accuracy (**b**) of the majority of the fish in the group to reach the arm with food, with different proportions of trained individuals. Note: a, b, c, d letters suggest a significant difference of the variables after different days of training (*p* < 0.05).

The speed with which the majority of the fish arrived at the arm with food decreased gradually and significantly with an increasing proportion of trained fish (Figure 3a) (*p* < 0.05). The among-group variation also decreased profoundly, as can be seen from the S.E. of each value. The accuracy rate of the majority, for first entering the arm with food rather than the arm without food, increased in line with the proportion of trained fish (y = 0.808x + 20.032, R^2^ = 0.970, *p* < 0.001), from 12.5% in the 0:8 group to 100% in the 8:0 group (Figure 3b).

## 4. Discussion

### 4.1. Learning and Memory Abilities of Cichlids

The experimental fish showed learning ability through an association between a landmark and food; the speed and accuracy of cichlids entering the arm with food improved profoundly with an increase in training time. The speed and accuracy improved steadily over the first five days of training, suggesting that such operant conditioning could be reinforced by increased training repetition. Similar results have been documented in fish species such as zebrafish [36]. However, no further increase in either speed or accuracy was noted from five to seven days, suggesting there is a threshold on this cognitive task. The speed and accuracy rose with the training process and finally reached a relatively stable threshold [37]. Eventually, the time required to find the arm with food shortened by 59.30% (i.e., from 22.53 s to 9.17 s), while the accuracy increased by 50.65% (i.e., from 44.25% to 66.67%). Notably, the improvement in speed was far more profound than that of accuracy. A possible reason for a more profound change in speed might be that the experimental fish behaved more bravely as they became familiar with the arena and training process. It is worth pointing out that the 40% accuracy on the first day is far higher than our theoretically calculated value (i.e., one-sixth, 13.3%). This suggests that cichlids prefer to enter the arm with plastic plants even without food information, possibly as plants act as shelter in natural habitats [38]. Thus, the accuracy rate had less room for improvement during the training process. Although this preference does have an influence, it has no effect on our experimental results. With the increase in training days, the association is still reinforced as the speed of individual fish becomes faster and faster and the accuracy becomes higher and higher.

The results of the memory retention test in the present study showed that cichlids can hold on to cognitive ability throughout training for at least 11 days, as no difference was found in either speed or accuracy after 2 to 11 days of detraining. Similar results have been documented in other animals, including fish species. Researchers have proposed that birds and mammals can retain their learning and memories for several months to years [39,40,41]. There are also extensive examples showing that the learning and memory capabilities of fish are impressive [42]. It has been found that gobies (*Bathygobius soporator*) can retain their memory for 40 days [43], while tilapia (*Sarotherodon galilaeus*) retain their memory of sound stimuli for up to six months [44]. Some researchers have suggested that cleaner fish (*Labroides dimidiatus*) can remember events such as being captured by nets for at least 11 months [45]. During their lives, fish will face many situations. They need to choose the most effective way to make use of their living environment [46], learn to explore and identify high-quality food resources [47], resolve conflicts with competitors, find suitable mates, and avoid being tracked by enemies [48]. All these require them to make appropriate responses to the situation at such times. Learning and memory play an important role in these behaviors of fish; therefore, the abilities of both learning and memory are consistent with fish’s adaptation to the environment and meeting their survival and reproduction needs.

### 4.2. Effects of Group Composition on Foraging Performance and the Possible Decision-Making Mechanism

The results showed that the speed and accuracy of the majority of the fish group entering the arm with food increased in line with an growing proportion of trained group members. This suggests that the behavior of the cichlid group might be decided equally by the group members, i.e., cichlids might adopt democracy in group decision making under a foraging context. Cichlids are a social species, living in high-predation habitats. A previous study found that the decision making of fish populations from high-predation habitats is more likely to be equally determined [49]. However, contrary to our expectations, the present study found that cichlids’ group foraging takes longer than that of individuals during the training process. The study suggests that behavioral interaction among group members might not always lead to an increased foraging efficiency in cichlids. Thus, the linear increase in performance with an increase in the number of trained fish, estimated by the majority of a group, in either speed or accuracy might be possible due to the fact that trained fish will be in the majority. Identifying each fish member to acquire the trajectory of each trained and nontrained group member in future can help better understand how the composition affects the group decision [50]. Nevertheless, the linear relationship between group composition and performance in the present study suggests that the group performance of cichlids might not be determined by a minority of group member with a specific threshold in a foraging context. It is worth noting that, by using the same six-arm maze, our observation found that time for the majority of the cichlid group to enter an arm with shelter after a simulated predator stimulation was much shorter than those of individual cichlids. This suggests that an ecological context other than foraging might be more appropriate to study the collective-decision mechanism for cichlids.

In conclusion, the present study found that cichlids show an impressive associative learning ability when performing a foraging task in a six-arm maze. Such cognitive ability might be ecologically important for cichlids living in complex habitats with high heterogeneity of food abundance. The foraging behavior of cichlids in the group seems unlikely be determined by a minority of group members in a foraging context. Further studies in other ecological contexts might yield interesting results.

## Figures and Tables

**Table 1:** Results of the memory retention test, i.e., comparison of speed and accuracy rate after 7 d of training with those for the same fish following a different period of detraining based on a linear mixed-model analysis (parameter data are presented as means S.E.).

Detraining Period		2 d	5 d	8 d	11 d	Statistical Analysis		
						Treatment Effect	Period Effect	Interaction Effect
Speed (s)	Training	7.33 ± 0.94	8.86 ± 2.54	8.17 ± 0.84	8.06 ± 0.92	*F*_1,43_ = 0.297 *p* = 0.589	*F*_3,43_ = 0.912 *p* = 0.175	*F*_3,43_ = 0.506 *p* = 0.680
	Detraining	10.82 ± 6.18	6.42 ± 1.56	6.42 ± 1.38	5.73 ± 1.39	*F*_3,42_ =0.164 *p* = 0.920	*F*_3,42_ = 0.473 *p* = 0.703	*F*_3,42_ = 0.473 *p* = 0.703
Accuracy rate (%)	Training	50 ± 0.15	69.44 ± 0.14	55.56 ± 0.15	69.44 ± 0.13	*F*_1,86_ = 1.691 *p* = 0.197	*F*_3,86_ = 0.349 *p* = 0.790	*F*_3,86_= 1.237 *p* = 0.301
	Detraining	61.11 ± 0.14	69.44 ± 0.14	55.56 ± 0.15	66.67 ± 0.14	*F*_3,42_ = 0.564 *p* = 0.642	*F*_3,42_ = 0.780 *p* = 0.512	*F*_3,42_ = 0.113 *p* = 0.593

## Data Availability

Data are available upon request from the corresponding author.
